# Expression of Toll-like Receptors in Stem Cells of the Apical Papilla and Its Implication for Regenerative Endodontics

**DOI:** 10.3390/cells12202502

**Published:** 2023-10-21

**Authors:** Koyo Takimoto, Matthias Widbiller, Anibal Diogenes

**Affiliations:** 1 Department of Endodontics, University of Texas Health Science Center at San Antonio, 7703 Floyd Curl Drive, San Antonio, TX 78229-3900, USA; takimotokouyou@gmail.com (K.T.); matthias.widbiller@klinik.uni-regensburg.de (M.W.); 2 Department of Conservative Dentistry and Periodontology, University Hospital Regensburg, 93053 Regensburg, Germany

**Keywords:** regenerative endodontics, TLR, innate response, stem cell, SCAP, apical papilla, odontoblastic, mineralization, chemotaxis

## Abstract

Regenerative therapies to replace cells and tissues damaged due to trauma and dental infections require temporal and spatial controlled recruitment and the differentiation of progenitor/stem cells. However, increasing evidence shows microbial antigens can interfere with this process. Toll-like receptors (TLRs) are crucial in recognizing pathogen-associated molecular patterns. Stem cells of the apical papilla (SCAP) are required for normal dental development and are intimately involved in the reparative and regenerative capacity of developing teeth. We hypothesized that TLRs are expressed in SCAP and that the activation of TLR2/TLR4 or TLR3 by different ligands results in differential cellular fate, impacting their differentiation into a mineralizing phenotype. We found that most TLRs are expressed as detected by PCR except TLR7 and TLR8; exposure to heat-killed E. coli results in upregulating TLR2 and TLR4 and reducing mineralization capacity. In addition, bacterial exposure resulted in the upregulation of 11 genes, of which 9 were chemokines whose proteins were also upregulated and released, promoting in vitro macrophage migration. On the other hand, TLR3 activation resulted in increased proliferation and a dramatic inhibition of osteogenic and odontoblastic differentiation, which was reversed by inhibition or the knockdown of TLR3 expression. The profound effects of TLR activation resulting in different cell fates that are ligand and receptor-specific warrants further evaluation and represents an important therapeutic target to make regenerative approaches more predictable following dental infections.

## 1. Introduction

The oral cavity has remarkable regenerative potential due to rich and distinct populations of mesenchymal stromal cells (MSCs). The dental pulp is designed to respond to various insults such as caries and trauma through a robust immunological response but also through the recruitment and activation of various stem cell niches to participate in the modulation of the immune responses and the reparative process. The apical papilla stem cells (SCAP) are contained within the dental apical papilla, a dense reservoir of undifferentiated MSCs, and have great proliferative and odontogenic differentiation capacity [1,2]. These cells are responsible for tooth development through their concerted interaction with epithelial cells from Hertwig’s root sheath [3]. These complex epithelial–mesenchymal interactions dictate root development and shape. Unfortunately, dental infections can lead to severe dental pulp injury, overwhelming its inherent regenerative potential, and ultimately resulting in arrested development and loss of function (i.e., tooth loss).

Microbial infections trigger a robust immunological response in the dental pulp [4,5]. The recognition of pathogen-associated molecular patterns (PAMPs) in dental infections by pathogen pattern recognition receptors (PRRs), such as Toll-like receptors (TLRs), represents one of the first steps in the defense response. A total of 10 different TLRs have been identified in human cells [6] with distinct signaling pathways and the ability to detect specific microbial “molecular signatures” such as lipopolysaccharides, flagellin, microbial DNA or RNA, but also few identified endogenous ligands [7,8]. It has become clear that these receptors are expressed in multiple cell types in addition to immune cells, including odontoblasts [9], dental fibroblasts [10], and stem cells [11]. Among these, the TLR4 receptor detects lipopolysaccharides (LPSs) derived from the surface of Gram-negative bacteria [12,13]. TLR2 recognizes lipoteichoic acid (LTA) from Gram-positive bacteria [14] and TLR3 double-stranded RNA from viruses [15] but is also shown to be activated by endogenous RNA [16]. These receptors have been reported in oral stem/progenitor cells, including dental pulp stem cells (DPSCs), periodontal ligament stem cells (PDLSCs), and SCAP. 

Regenerative endodontic procedures rely on the surgical transfer of stem cells from the apical tissues by the laceration of apical tissues and intracanal bleeding [17]. Stem cells of the apical papilla are believed to be the primary cell type involved in currently employed regenerative procedures since they have been found to survive advanced apical periodontitis following infection and pulpal necrosis [18] and demonstrate high proliferative and differentiation potential in hypoxic environments [19]. Furthermore, pulpal infection becomes increasingly rich with Gram-negative obligate and facultative anaerobes as it progresses through the canal system. Thus, SCAP are undoubtedly exposed to bacterial antigens and possibly endogenous RNA released from damaged cells. However, the differential effect of these microbial ligands on SCAP differentiation fate is poorly understood. Thus, this study aimed to evaluate the expression of Toll-like receptors in SCAP and the effect of bacterial antigens or RNA-like ligands on proliferation and differentiation into mineralizing or immunomodulatory cells.

## 2. Materials and Methods

### 2.1. SCAP Culture

A previously characterized SCAP cell line was used in all experiments [20]. Briefly, cells were cultured at 37 °C in 5% CO_2_ in media comprised of alpha-modified minimum essential medium (α-MEM; Sigma Aldrich, St. Louis, MO, USA) containing 10% heat-inactivated fetal bovine serum (FBS; Gibco, Life Technologies, Grand Island, NY, USA) and 1% glutamine/penicillin/streptomycin solution (Gemini Bio-Products, West Sacramento, CA, USA). Upon reaching 80% confluency, cells were passed onto other cell culture flasks following trypsinization and used in subsequent experiments, or the media switched to osteogenic media composed of the media described above supplemented with differentiation factors (0.5% ascorbic acid and 1% β-glycerol phosphate) at 37 °C and 5% CO_2_ for 14 days for differentiation into a mineralizing phenotype.

For all subsequent experiments, cells were cultured in basal media or under osteogenic differentiation and exposed to Toll-like receptor ligands, LPSs (0.01 µg/mL or 0.1 µg/mL), heat-killed *E. coli* (1 × 10^17^ cells/mL), or poly (I:C) (0.1 µg/mL or 1 µg/mL) in the presence or absence of 10 µM of CU CPT4a (TLR3 inhibitor; Tocris, Minnesota, MN, USA).

### 2.2. Immunocytochemistry

Cultured SCAP were processed as described previously [21]. Briefly, all cells were fixed with 4% paraformaldehyde for 1 h at room temperature and washed 3 times for 10 min in phosphate-buffered saline (PBS) (Sigma). Next, cells were permeabilized and blocked for nonspecific protein binding sites with a blocking solution consisting of 4% normal goat serum (Sigma), 2% bovine gamma-globulin (Sigma), and 0.3% Triton X-100 (Thermo Fisher Scientific, Rockford, IL, USA) in PBS for 60 min before incubation overnight with mouse antibodies against human TLR4 (1:250) (Abcam, Waltham, MA, USA) or TLR3 (1:200) (Abcam), which was followed by staining for the cytoskeleton protein phalloidin (Alexa Fluor Phalloidin 568; Thermo Fisher Scientific) and the nuclear stain DAPI (Thermo Fisher Scientific). Immunoreactivity was visualized with anti-mouse Alexa Fluor 488 secondary antibodies (1:200; Thermo Fisher Scientific). Immunoreactivity was evaluated with an EVOFL inverted microscope (Life Technologies; Carlsbad, CA, USA). Controls consisted of evaluating cells that were stained as described above but lacked primary antibodies.

### 2.3. Western Blot

Total protein was extracted from SCAP exposed to either vehicle or heat-killed *E. coli* in NP-40 buffer in the presence of protease inhibitors (Roche, Indianapolis, IN, USA). Approximately 20 µg of protein samples per lane was resolved on 12.5% SDS-PAGE and transferred to PVDF (Millipore, Billerica, MA, USA), and the Western blots were blocked in 5%BSA in TBS-Tween and visualized using antibodies to TLR2 (1:200, Abcam) TLR3 (1:200, Abcam), TLR4 (1:200, Abcam) or GAPDH (1:1000, Abcam). 

Autoradiography and Western blot results were scanned and quantified using the ChemiDoc digital documentation cabinet (Bio-Rad, Hercules, CA, USA). All autoradiographic bands were normalized to values of GAPDH. Results are representative of 3 independent experiments.

### 2.4. Proliferation Assay

Stem cells of the apical papilla were plated at 1 × 10^4^ cells/well and cultured in 24-well plates for 1, 4, and 7 days in the presence of vehicle, LPS. Then, the quantity of viable cells was determined using CellTiter-Glo reagent (Promega, Madison, WI, USA) with an incubation at room temperature for 10 min. Blank luminescence was used for calibration of the assay. A luminescence plate reader, the FlexStation 3 Benchtop Multi-Mode microplate reader (Molecular Devices, San Jose, CA, USA), was used to determine the relative values for each group.

### 2.5. Quantitative Mineralization Assay

For the quantification of mineralization potential, SCAP were cultured in osteogenic differentiation media for 21 days with media changed every 3 days in the presence of either vehicle, ultra-pure *E. coli* LPSs (0.01 µg/mL, 0.1 µg/mL) (Invivogen, San Diego, CA), poly (I:C) 0.1 µg/mL) or heat-killed *E. coli* (1 × 10^17^ cells/mL). At the end of the culture period, calcium deposits within cells and the extracellular matrix were first visualized under 10× magnification brightfield microscopy using an EVOS FL microscope (Life Technologies). Next, mineralization was quantified by staining using the osteogenesis quantification kit (Millipore, Darmstadt, Germany). Briefly, all cells were fixed with 4% paraformaldehyde for 1 h at room temperature, washed 3 times for 10 min in PBS, and stained with Alizarin red; then, absorbance was measured at 405 nm using a multimode FlexStation 3 Benchtop Multimode Microplate Reader.

### 2.6. RT-PCR 

Cultured SCAP were washed in phosphate-buffered saline and lysis buffer from a RNeasy Plus Mini Kit (Qiagen, Hilden, Germany) added to the cells. Total RNA was isolated according to the manufacturer’s instructions. cDNA was synthesized using the Applied Biosystems High-Capacity RNA-to-cDNA Kit (Thermo Fisher Scientific, Waltham, MA, USA). Semi-quantitative polymerase chain reaction (PCR) was performed using primers specific to *TLR1* through *TLR10* (Human TLR PCR panel, Invivogen) and a PCR master Mix (Promega) with reactions run in an Applied Biosystems MiniAmp Thermocycler (Thermo Fisher Scientific), which was followed by electrophoresis in 1% Agarose (Bio-Rad, Hercules, CA, USA) and imaging using the ChemiDoc digital documentation cabinet (Bio-Rad). Real-time RT-PCR reactions for the following targets: dentin sialophosphoprotein (*DSSP*, assay Hs00171962_m1), alkaline phosphatase (*ALP*, assay Hs03046558_s1), *TLR3* (assay Hs01551079_g1) and *18S* (assay #Hs99999901_s1) were performed using the TaqMan Fast Advanced Master Mix (Thermo Fisher Scientific), and amplification was performed on an ABI7500 Fast Real-Time PCR System (Thermo Fisher Scientific). Expression fold change was determined using the comparative delta–delta cycle threshold method (ΔΔCt) after normalization to the endogenous control expression using the control group as a reference sample.

### 2.7. RT-PCR Array 

Total RNA samples isolated from SCAP exposed to either vehicle or heat-killed *E. coli* (*n* = 3 biological replicates/group) were used to synthesize cDNA templates as described above and used in PCR reactions using the RT² Profiler™ PCR Array Human Inflammatory Response & Autoimmunity (Qiagen, GeneGlobe ID—PAHS-077Z) following the manufacturer’s instructions. All reactions were performed on an ABI7500 Fast Real-Time PCR System (Thermo Fisher Scientific), and exported cycle threshold values were uploaded onto the GeneGlobe analysis web-based tool (https://geneglobe.qiagen.com/us/analyze, accessed on 9 August 2023) to yield differentially expressed genes with fold >2 and *p* < 0.05. 

### 2.8. TLR3 siRNA 

SCAP were incubated with either an oligo Silencer^®^ against the human *TLR3* gene (Thermo Fisher Scientific; assay #107054) or a scrambled control Silencer^®^ Control 1 (Thermo Fisher Scientific; assay #AM4611) in the presence of Lipofectamine RNAiMAX transfection reagent (Thermo Fisher Scientific) in culture media and conditions described above for 3 days, which was followed by a media change and subsequent experiments. 

### 2.9. Multiplex Analysis of Inflammatory Mediators

Conditioned media was collected once every 2.5 days from SCAP cultured in the presence of a vehicle or heat-killed *E. coli* for 5 days (*n* = 9 biological replicates/group). The media were immediately frozen at −80 °C and stored until assayed. We used Luminex xMAP technology to quantify 64 cytokines, chemokines, and growth factors in condition media. The multiplexing analysis was performed using the Luminex™ 100 system (Luminex, Austin, TX, USA) by Eve Technologies Corp. (Calgary, AB, USA). All 64 inflammatory markers were measured in cell culture media samples using MILLIPLEX Human Cytokine/Chemokine Discovery 23 and 41-plex kits (Millipore, St. Charles, MO, USA) according to the manufacturer’s protocol. The assay sensitivities of the 64-plex markers range from 0.1 to 7 pg/mL on average. Targets with detectable levels with fold change > 2 and *p* < 0.05 were included in the analysis. 

### 2.10. Transwell Migration Assay

Murine RAW264.7 cells (ATCC; Manassas, VA, USA) were preloaded with Vybrant™ Di-I Cell-Labeling Solution (Thermo Fisher Scientific) according to the manufacturer’s instructions. Approximately 5 × 10^4^ cells/insert were placed in FluoroBlok™ 24-well cell culture light-blocking inserts with 8 µm pores (Corning, Corning, NY, USA) and allowed to equilibrate in normal culture media and conditions described above for 6 h. SCAP were cultured as described above at the concentration of 1 × 10^5^ cells/well in black-walled 24-well plates (Corning) in the presence or absence of 1 × 10^17^ heat-killed bacteria. After 2 days of culture, inserts containing the labeled macrophage cell line (RAW264.7) were transferred to the SCAP plates. After 24 h of co-culture, the fluorescence of the lower chamber was measured at 480 nm using a FlexStation 3 Benchtop Multi-Mode microplate reader (Molecular Devices, San Jose, CA, USA); then, representative images of fluorescently labeled cells were acquired using an EVOFL inverted microscope (Life Technologies) at 10× magnification. 

### 2.11. Statistical Analysis 

Data were subjected to Student’s *t*-test, one-way or two-way analysis of variance (ANOVA) followed by the Bonferroni’s post-hoc test. Statistical analysis with significant values set at *p* < 0.05 was tested using the GraphPad Prism version 6.1 software (GraphPad, La Jolla, CA, USA). 

## 3. Results

### 3.1. Toll-like Receptors Expression in Stem Cells of the Apical Papilla (SCAP)

**Figure 1:**
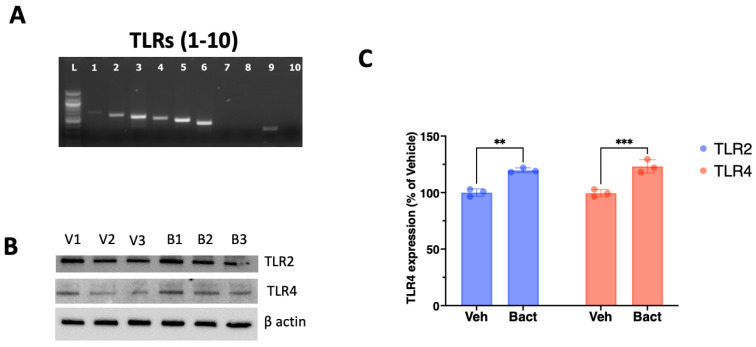
Differential representation of Toll-like receptors in SCAP. (**A**) Stem cells of the apical papilla express the genes for *TLR1*, *2*, *3*, *4*, *5*, *6* and *9* as detected by RT-PCR. (**B**) The expression of TLR 2, 3 and 4 was detected by Western blot in SCAP exposed for 5 days to either vehicle (V1, V2 and V3) or heat-killed *E. coli* (B1, B2 and B3) in extracts from 3 independent experiments. (**C**) Exposure to heat-killed bacteria (Bact) significantly increased the expression of TLR2 and TLR4 compared to vehicle (Veh) after 5 days of exposure. ns = not statistically significant; ** = *p* < 0.01, *** *p* < 0.001; Student’s *t*-test.

The gene expression for *TLR1, TLR2, TLR3, TLR4, TLR5, TLR6* and *TLR9* was confirmed by RT-PCR (Figure 1A). Exposure to heat-killed (HK) *E. coli* increased the protein expression of *TLR4* (Figure 1B,C) by approximately 23% (*p* = 0.004) and *TLR2* by approximately 19% (*p* = 0.001) (Figure 1B,C). 

### 3.2. Effect of Lipopolysaccharides or Heat-Killed Escherichia Coli (HK E. coli) on SCAP Proliferation and Mineralization

**Figure 2:**
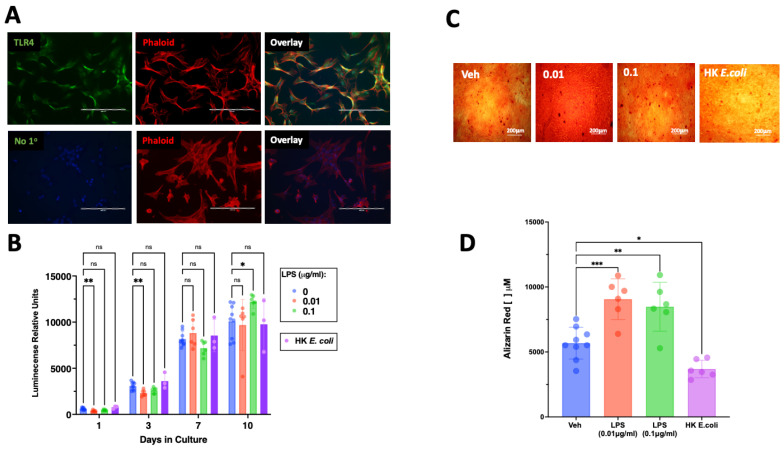
Expression of TLR4 in SCAP and its effect on proliferation and osteogenic differentiation. (**A**) TLR4 immunoreactivity is visualized in green and found in the majority of cultured SCAP, the cytoskeleton is visualized in red by staining of phalloidin (Phalloid). The staining for TLR4 was absent in samples lacking the primary antibody (No 1°). (**B**) Exposure to different concentrations of LPSs resulted in reduced proliferative capacity of SCAP, whereas exposure to heat-killed *E. coli* did not affect the number of viable cells over 10 days of culture. (**C**) Representative pictures of Alizarin red staining in SCAP cultured in osteogenic media in the absence of antigens (Veh), LPSs (0.01 µg/mL, 0.1 µg/mL), or heat-killed *E. coli*. (**D**) Exposure to LPSs at both tested concentrations for 21 days resulted in increased mineralization, whereas whole bacterial extracts resulted in reduced mineralization. ns = not statistically significant; * = *p* < 0.05; ** = *p* < 0.01; *** *p* < 0.001; two-way ANOVA (panel (**B**)) one-way ANOVA (panel (**D**)) with Bonferroni’s post hoc test. Scale bar = 200 μm.

Exposure of SCAP to LPSs led to an early decrease in cell proliferation detected at 1 and 3 days in culture and an increase in proliferation on day 10 for the highest LPS concentration tested (Figure 2B). Interestingly, exposure to heat-killed *E. coli* did not alter the number of viable cells detected in each time point (Figure 2B).

Exposure to LPSs for 21 days increased the differentiation into a mineralizing phenotype with greater mineralization detected (Figure 2C) and quantified (Figure 2D). Conversely, exposure to HK *E. coli* reduced mineralization (Figure 2C,D).

### 3.3. Exposure of SCAP to HK E. coli Results in Differentiation into an Immunocompetent Phenotype

**Figure 3:**
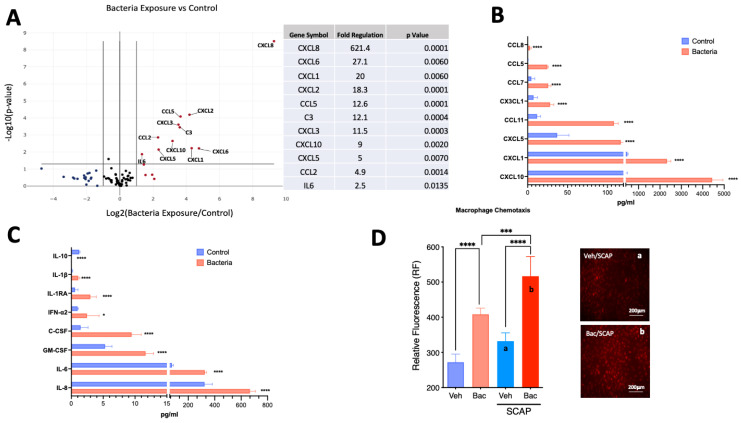
Bacterial antigens evoke the upregulation of pro-inflammatory markers in SCAP. (**A**) Volcano plot showing results from PCR array with the upregulation of 11 genes with greater than 2-fold change and *p*-value < 0.05 (*n* = 4/group). (**B**) Chemokine protein quantification by Luminex of targets upregulated in conditioned media by SCAP exposed to heat-killed *E. coli* for 5 days (*n* = 4/group). (**C**) Cytokine protein quantification by Luminex of targets upregulated in conditioned media by SCAP exposed to heat-killed *E. coli* for 5 days. (**D**) SCAP exposed to bacterial antigens for 5 days significantly increased the migration of fluorescently tagged macrophages in a Transwell assay. Representative images of groups (a) and (b) were acquired. * = *p* < 0.05; *** *p* < 0.001; **** = *p* < 0.0001, Student’s *t*-test for panels (**A**–**C**) and one-way ANOVA with Bonferroni’s post-hoc test.

Exposure to heat-killed *E. coli* (1 × 10^17^ cells/mL) for 5 days resulted in the significant upregulation of 11 genes, of which 9 were chemokines (*CXCL8, CXCL6, CXCL1, CXCL2*, *CCL5, CXCL3, CXCL10, CXCL5* and *CCL2*), followed by interleukin 6 (IL-6) and complement component 3 (*C3*) (Figure 3A).

### 3.4. Inflammatory Markers Protein Expression

Exposure to heat-killed *E. coli* (1 × 10^17^ cells/mL) for 5 days resulted in the upregulation of the following chemokines present in the media (Figure 3B): CXCL10 (11.6-fold, *p* < 0.0001); CXCL1 (4.9-fold, *p* < 0.0001); CXCL5 (3.15-fold, *p* < 0.0001); CCL11 (9.1-fold, *p* < 0.001); CXC3CL1 (3.8-fold, *p* < 0.0001); CCL7 (5.5-fold, *p* < 0.0001); CCL5 (112.7-fold, *p* < 0.0001); and CCL8 (3-fold; *p* < 0.001). In addition, there was a significant increase in the detection of the following cytokines and growth factors (Figure 3C): IL-8 (2-fold, *p* < 0.0001); IL-6 (4.2-fold, *p* < 0.0001); GM-CSF (2.2-fold, *p* < 0.0001); C-CSF (6.4-fold, *p* < 0.0001); IFN-α2 (2.5-fold, *p* < 0.05); IL-1RA (5-fold, *p* < 0.0001); IL-1β (7.4-fold, *p* < 0.0001). Conversely, IL-10 was the only target with a detected significant decrease (10-fold; *p* < 0.0001).

### 3.5. Macrophage Migration

To evaluate the functional activity of the detected chemokines, macrophages were co-cultured with SCAP exposed to either vehicle or heat-killed *E. coli* for 24 h. Macrophages migrated toward bacteria in the absence of SCAP (approximately 50% increase in migration, *p* < 0.0001 compared to vehicle control) (Figure 3D). However, the migration was significantly enhanced (*p* < 0.001) by co-culture with SCAP in the presence of bacteria (approximately 90% increase, *p* < 0.0001 compared to vehicle control).

### 3.6. TLR3 Activation Regulation of SCAP Proliferation and Differentiation

**Figure 4:**
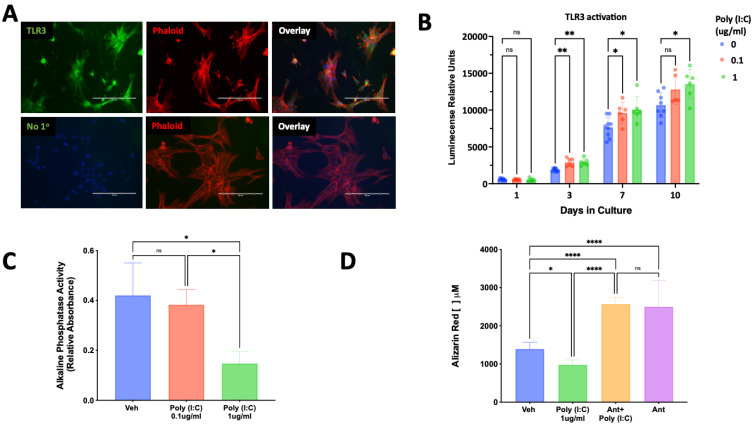
Expression of TLR3 and its effect on SCAP proliferation and osteogenic differentiation. (**A**) TLR3 immunoreactivity is visualized in green and found highly expressed in cultured SCAP, the cytoskeleton is visualized in red by the staining of phalloidin (Phalloid). The staining for TLR3 was absent in samples lacking the primary antibody (No 1°). (**B**) Poly (I:C) evoked a concentration and time-dependent increase in SCAP proliferation (*n* = 6–9/group/time). (**C**) Activation of TLR3 by poly (I:C) reduced alkaline phosphatase activity in a concentration-dependent manner (*n* = 6). (**D**) Activation of TLR3 by poly (I:C) resulted in reduced mineralization, which was reversed and increased by a TLR3 inhibitor detected by a quantitative Alizarin red assay. ns = not statistically significant; * = *p* < 0.05; ** = *p* < 0.01 and **** = *p* < 0.0001 by two-way ANOVA (panel **B**) and one-way ANOVA (panels **C**,**D**) with Bonferroni’s post-hoc test. Scale bar = 200 μm.

Exposure of SCAP to the TLR3 ligand poly (I:C) resulted in a concentration and time-dependent increase in SCAP proliferation (Figure 4B). Poly (I:C) evoked a decrease in alkaline phosphatase activity (Figure 4C) and mineralization in SCAP cultured under osteogenic induction (Figure 4D). The reduction in mineralization observed in SCAP cultured for 14 days in the presence of poly (I:C) was significantly reversed by the potent TLR3 inhibitor, CU CPT4a (Figure 4D).

**Figure 5:**
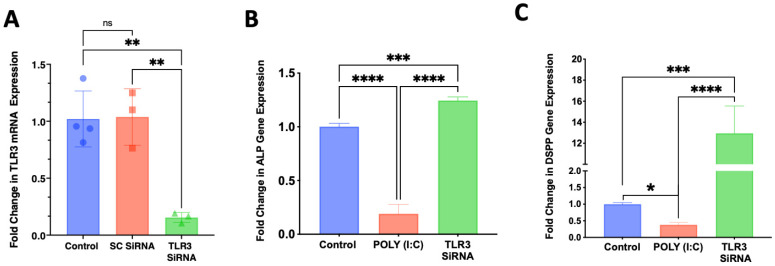
Knockdown of *TLR3* with SiRNA for 3 days reverses the inhibitory effects of poly (I:C) on differentiation into a mineralizing phenotype of SCAP culture in presence of differentiation media for an additional 7 days. (**A**) A designed siRNA against *TLR3* resulted in approximately 80% reduction in mRNA expression while a control siRNA (SC, scrambled sequence) had no effect (*n* = 3–4/group). (**B**) Knockdown of *TLR3* reversed the inhibition of *ALP* mRNA expression evoked by poly (I:C) (*n* = 4/group). (**C**) Knockdown of *TLR3* expression by siRNA reversed poly (I:C) inhibition and increased the expression of the odontoblast marker DSPP (*n* = 6/group). ns = not statistically significant; * = *p* < 0.05; ** = *p* < 0.01; *** = *p* < 0.001 and **** = *p* < 0.0001. one-way ANOVA with Bonferroni’s post-hoc test.

Treatment of SCAP with siRNA for 3 days resulted in the knockdown in *TLR3* gene expression, while the scrambled siRNA sequence had no effect (Figure 5A). This knockdown of *TLR3* reversed the inhibition of alkaline phosphatase (*ALP*) (Figure 5B) and dentin sialophosphoprotein (*DSPP*) gene expression evoked by poly (I:C) when SCAP were cultured in osteogenic media for an additional 7 days following TLR3 knockdown (Figure 5C).

## 4. Discussion 

Predictive pulpal repair and regeneration remain elusive despite significant advances in pulp biology and regeneration knowledge. An increasing body of evidence demonstrates the feasibility of pulpal regeneration, including the differentiation of stem cells into odontoblast-like cells expressing dentin sialophosphoprotein (DSPP). However, most studies focused on outcomes in sterile conditions while the damaged pulp is invariably exposed to a barrage of microbial antigens. The stem cells of the apical papilla (SCAP) are among the stem cells responsible for dental development. They are considered crucial in the repair and regeneration of the damaged pulp in immature teeth. In this study, we demonstrated that SCAP express most Toll-like receptors and that the activation of TLR2/4 leads to different cellular responses from the activation of TLR3.

We demonstrated that SCAP express TLR1, 2, 3, 4, 5, 6 and 9. The expression of Toll-like receptors has been directly investigated in other oral-derived mesenchymal stromal cell populations such as human periodontal ligament stem cells (hPDLSCs) [22], dental pulp stem cells (DPSCs) [23], human gingival mesenchymal stem cells (hGMSCs) [24], bone marrow mesenchymal stem cells (BM-MSCs) [25] and SCAP [26]. Notably, the TLRs’ expression profile varies within these different cell populations [27]. For example, the previously characterized SCAP cell line used in this study did not have any detectable levels of TLR7 or TLR8, while these receptors have been detected in SCAP in another study [26] but also in hPDLSCs [22]. Other studies have focused on the cellular response upon exposure to different ligands as evidence that a specific TLR is expressed and functional in these cells. We did not verify whether SCAP expressed TLR10, which is a less understood TLR, but it has been shown to trigger anti-inflammatory responses due to the production of IL-1Ra [28] but also pro-inflammatory if participating in the recognition of *H. pylori* LPSs in conjunction with TLR2 [29]. Despite the robust expression profile of most TLRs in SCAP, we focused on the differential function of TLR2/TLR4 versus TLR3 activation, since these receptor systems are the most likely activated upon microbial infections in the dental pulp that are predominant with Gram-positive and -negative bacteria [30], possibly viruses [31] and endogenous mRNA released upon cell death.

The robust expression of TLR2 and TLR4 in SCAP agrees with the expression of these essential bacterial recognition receptors in other oral-derived MSCs such as DPSCs [23] and the functional effects of their ligands upon cellular fate [32,33,34]. We found that exposure to heat-killed *E. coli* upregulated the protein expression of TLR4 with no effect on the expression levels of TLR2 or TLR3. In this study, we used heat-killed *E. coli* as potent TLR2/TLR4 ligands [35], mimicking a bacterial infection known to have an entourage of bacterial ligands and not just LPSs or LTA [36]. Nonetheless, we demonstrated that exposure of SCAP to LPSs resulted in a concentration- and time-dependent decrease in cell proliferation while increasing differentiation into a mineralizing phenotype seen as a greater detection of calcium deposits by Alizarin red. The reduction in proliferation is consistent with the cells entering a differentiation stage resulting in increased mineralization. Although heat-killed *E. coli* also resulted in a reduction in cellular proliferation, it did not increase mineralization; instead, it resulted in a robust decrease in mineralization. This finding supports that complex microbial antigens can result in a differential effect on stem cell fate that is not necessarily seen with the exposure of highly purified single ligands such as LPS. Also, SCAP in the presence of HK bacteria appeared to have differentiated into an immunomodulatory fate instead of a mineralizing phenotype seen in the robust upregulation of chemokine and cytokine expression. We have previously demonstrated that exposure to a single-species bacterial biofilm significantly reduced the dentinogenic differentiation potential of SCAP [37]. Moreover, secreted by-products of oral bacteria in biofilms can differentially regulate the mineralization of SCAP in a bacterial species-specific manner [38]. Thus, the findings of this study and others suggest that the differential activation of TLRs by a myriad of bacterial antigens profoundly affects the differentiation potential of MSCs. This has profound implications for the regeneration of damaged mineralized tissues, which relies on appropriate differentiation, with spatial and temporal control, into an odontoblast-like or cementoblast-like cell phenotype that may be impeded by residual microbial antigens after disinfection. 

The ability of MSCs to modulate the microenvironment has been long appreciated. In general, it is believed that MSCs have a robust anti-inflammatory effect. This is partly due to the study paradigm in which these progenitor cells are exposed to a wide range of inflammatory mediators or placed within an inflammatory site in vivo [39,40]. In this study, SCAP exposed to heat-killed bacteria responded with a robust upregulation of pro-inflammatory cytokines such as complement *C3* and *IL-6* at the gene expression levels but a robust upregulation of gene expression of several chemokines, including the members of the CXC chemokine family (*CXCL3, CXCL5, CXCL6, CXCL8* (*IL-8*), and *CXCL10*) and other chemokines such as *CCL5* and *CCL2*. Furthermore, SCAP demonstrated a robust production and release of chemokines into the media detected by multiplex luminescence assays. The release of the anti-inflammatory cytokine IL-10 was reduced upon bacterial exposure, further demonstrating that the cells acquired a pro-inflammatory profile. The inflammatory mediator that increased the most at both the gene and protein levels was *CXCL8*, also known as *IL-8*, which is crucial in neutrophil and M1 macrophage recruitment [41]. Also, CXCL10, CCL5, and CCL7 are all known to evoke the robust chemotaxis of pro-inflammatory macrophages and were robustly upregulated upon bacterial exposure [42]. We further demonstrated that these SCAP-released chemokines were functional by an in vitro macrophage migration assay. RAW264.7 were exposed to heat-killed bacteria and demonstrated innate migration toward the antigens within 24 h, significantly increasing in the presence of SCAP, demonstrating that release soluble factors were robustly recruiting macrophages. Thus, exposure of SCAP to bacteria evoked a shift to a pro-inflammatory phenotype which favored the expression and release of chemokines responsible for attracting cells of the innate immune response. These data demonstrate that SCAP are not simply downregulating an inflammatory response. Instead, they are active participants in the recruitment of immune cells through chemokines and the expression of key inflammatory mediators known to be significant participants in response to dental infections such as IL-6 and IL-1α [43]. Lastly, this robust employment of cellular machinery for producing and releasing inflammatory mediators likely prevents the cells from acquiring the reparative or regenerative phenotype of mineralizing cells. 

We demonstrated that TLR3 is broadly expressed within the cytoplasm of SCAP. The role of TLR3 in oral MSCs is largely unknown, as these receptors are best known for recognizing double-stranded viral RNA [15], and these viruses (e.g., rotaviruses) are not known pathogens in the infected dental pulp [44]. However, an increasing body of evidence suggests that dsRNA is not restricted to certain viruses and can be generated endogenously by eukaryotic cells due to cellular dysregulation in various pathophysiological conditions such as the accumulation of transposable elements, changes in RNA synthesis and processing, and mitochondrial damage [16]. Exposure of SCAP to poly (I:C), a potent RNA mimetic TLR3 ligand, resulted in a concentration and time-dependent increase in proliferation, resulting in a marked decrease in mineralization activity reversed by a TLR3 inhibitor. This differentiation into a mineralizing phenotype reduction was accompanied by reduced alkaline phosphatase (ALP) mRNA expression and activity, normally elevated in mineralizing cells, and the profound downregulation of *DSPP* expression, which is a marker of odontoblastic differentiation. Interestingly, the knockdown of *TLR3* expression in SCAP resulted in the reversal of ALP mRNA expression inhibition and the robust increase in DSPP to levels greater than control untreated cells. Since the odontoblastic differentiation of SCAP has long been elusive, particularly in the presence of infection, this is the first line of evidence that suggests that TLR3 is involved in odontoblastic differentiation. Activating TLR3 appears to be a molecular “trigger” released from damaged cells for progenitor proliferation from stem cell niches to prepare for repair and regeneration. 

## 5. Conclusions 

Collectively, this study demonstrates the expression of Toll-like receptors in SCAP, which is an important stem cell type for dental regeneration, and that TLR2/TLR4 activation with bacterial antigens shifts cells into a pro-inflammatory phenotype primarily dedicated to the recruitment of immune cells through the robust release of a wide range of chemokines. Moreover, activation of the highly expressed TLR3 signals cells to proliferate and profoundly inhibits differentiation into a mineralizing phenotype, particularly inhibiting the marker for odontoblast-like cells (DSPP). The regulation of stem/progenitor cells by TLR ligands, either through highly conserved microbial antigens or endogenous sources, warrants further investigation and may represent a significant therapeutic target for regenerative therapies following dental infections. 

## Data Availability

Data are available from the corresponding author.
